# Current lung cancer screening guidelines may miss high-risk population: a real-world study

**DOI:** 10.1186/s12885-020-07750-z

**Published:** 2021-01-11

**Authors:** Guiyi Ji, Ting Bao, Zhenzhen Li, Huairong Tang, Dan Liu, Ping Yang, Weimin Li, Yan Huang

**Affiliations:** 1 Health Management Center, West China Hospital, Sichuan University, Chengdu, Sichuan China; 2 Division of Pulmonary and Critical Care Medicine, West China Hospital, Sichuan University, Chengdu, Sichuan China; 3 Division of Epidemiology, Department of Health Sciences Research, Mayo Clinic, Rochester, MN USA; 4 Department of Health Sciences Research, Mayo Clinic, Scottsdale, AZ USA; 5 Precision Medicine Research Center, West China Hospital, Sichuan University, Chengdu, Sichuan China; 6 Frontiers Science Center for Disease-related Molecular Network, Sichuan University, Chengdu, Sichuan China; 7 The Research Units of West China, Chinese Academy of Medical Sciences, West China Hospital, Chengdu, Sichuan China

**Keywords:** Lung cancer, Screening, High-risk population, Health examination population, Real-world study, LDCT

## Abstract

**Background:**

Despite much research published on lung cancer screening, China has had no large-scale study on the missed diagnosis of lung cancer in a health examination population. We therefore did a real-world study using the current lung cancer screening guidelines to a health examination population in China to determine the proportion of lung cancer cases that have been missed.

**Methods:**

A real-world cohort study of screening, with the use of low-dose computed tomography, was conducted among people who took yearly health checkup in health management center of West China Hospital between 2006 and 2017. We respectively used current guidelines including lung cancer screening guidelines of the U.S. Preventive Services Task Force (USPSTF) and expert consensus on low dose spiral CT lung cancer screening in China.

**Results:**

In a total of 15,996 participants with health examination who completed the baseline screening, 6779 (42.4%) subjects had at least one positive finding, and 142 (2.1%) cases of lung cancer were screened positive. The false positive rate was 97.9%. Of 142 lung cancer cases detected in our study, only 9.2% met the lung cancer screening guidelines proposed by the USPSTF, and 24.4% met that of China. The rates of missed diagnosis were as high as 90.8 and 75.6% respectively. In addition, we did an in-depth analysis by gender. We found that among male patients with lung cancer, the proportion of smokers was 75%, and the proportion of young people under 50 was 23.2%. Among female patients with lung cancer, the proportion of smokers was only 5.8%, and the proportion of young people under 50 was up to 33.3%.

**Conclusions:**

The rate of missed diagnosis was as high as 90.8% applying the current lung cancer screening guidelines to the health examination population in China. Further study to determine screening guidelines for targeted populations, is warranted.

## Background

Lung cancer is the most frequent cancer and the leading cause of cancer death all over the world [1]. It is reported that the survival rate of lung cancer was negatively correlated with the clinical and pathological stages at the time of diagnosis [2]. Unfortunately, when symptoms appear, the disease is usually advanced and incurable [3]. The study showed that low-dose computed tomography (LDCT) can detect some tumors at early stages [4]. Many authoritative medical organizations have launched screening guidelines, which recommended lung cancer screening in high-risk groups with LDCT [5–7].

Generally, age and smoking are the main criteria to define high-risk groups in domestic and foreign guidelines. However, researches showed that except for smoking, the causes of lung cancer were also attributed to air pollution, environmental exposure, genetic factors [8, 9]. Although most lung cancers are caused by smoking, a lot of lung cancer cases worldwide have been reported in non-smokers [8]. If lung cancer in never smoker was considered as a separate category, it would be the seventh most common cause of cancer death around the world [10]. In addition, the incidence of lung cancer also shows a trend of youth in recent years [11–13]. Young patients with lung cancer have gradually become a disease group that can not be ignored [11–13].

For these reasons, the current lung cancer screening guidelines may miss some persons at high risk of lung-cancer especially young or non-smoking people [5–7, 14, 15]. However, there is still a lack of large-scale research on this in China. Therefore, in order to determine whether the current screening guidelines of lung cancer will lead to miss diagnosis of lung cancer cases in China, and to determine the fraction of lung cancer cases that would be missed, we did a real-world study which applied these screening guidelines to the health examination population in West China Hospital.

## Methods

### Trial oversight

We applied the current guidelines to the health examination population and compared them with the real world. This research, a study of screening with the use of LDCT, was conducted among the people taking yearly health checkup in health management center of West China Hospital.

### Participants

Participants were enrolled and screened from June 30, 2006 through June 30, 2017. They were followed for events that occurred before December 31, 2017.

Eligible subjects did not have undergone chest imaging within 18 months before enrollment, and there were no new or aggravating cough, expectoration, hemoptysis, chest distress, dyspnea and other symptoms. Persons with any of the following conditions were excluded from this study: 1) previously received a diagnosis of unknown pulmonary nodules or malignant pulmonary nodules, masses, hilum enlargement, atelectasis; 2) a history of total or partial lobectomy; 3) history of lung cancer; 4) an unexplained weight loss of more than 5 kg in the past year. A total of 15,996 persons were enrolled. A written informed consent was obtained from every participant. Eligible participants completed a questionnaire that covered some topics, including demographic characteristics, smoking behavior, and medical history.

### Guidelines

In this study, we respectively used two kinds of the current lung cancer screening guidelines including lung cancer screening guidelines of the U.S. Preventive Services Task Force (USPSTF) and expert consensus on low dose spiral CT lung cancer screening in China to the health examination population in China to determine the proportion of lung cancer cases that have been missed. The USPSTF recommended annual screening for lung cancer with LDCT in adults aged 55 to 80 years, who currently smoke or have quit within the past 15 years, and who have an at least 30 pack-years of cigarette smoking history [5, 6]. The consensus of Chinese experts suggest that annual LDCT lung-cancer screening for individuals aged 50–75 years, combining at least one of the following risk factors: 1) at least 20 pack-years of cigarette smoking history, including currently smoking or giving up smoking for less than 15 years; 2) passive smoking; 3) a history of occupational exposure, including asbestos, beryllium, uranium, radon, etc.; 4) a history of cancer or a family history of lung cancer; and 5) a history of chronic obstructive pulmonary disease (COPD) or diffuse pulmonary fibrosis [7].

### Screening

Participants were invited to undergo a base-line screening. We conducted annual screenings from the next year. Participants with positive screening would be followed up, and those with negative screening would be screened in the next round.

All screening tests were conducted in accordance with a standard protocol developed by the medical physicists associated with the trial, which specified the acquisition variables and the acceptable characteristics of the machine [16–18]. All computed tomography (CT) scans were performed on double row spiral CT (Somatom Emotion Duo, Siemens, Germany). Thin slice scanning with 1 mm was performed on the local lesions. All scans were obtained using a low-dose regimen, with the machine set at 120 kVp, 16 (20 mA/0.8 s) ~ 40 (50 mA/0.8 s) mAs, pitch ≤1 cm, and 0.8 s rotation time. Chest radiographs were obtained with the use of digital equipment. All the machines used for screening met the technical standards [4].

Radiologic technologists and radiologists were certified by appropriate agencies. Radiologic technologists completed training in image acquisition. Radiologists also completed training in standardized image interpretation. Two radiologists with at least 5 years of experience in thoracic radiology assessed all chest images independently. Images were assessed first in isolation and then in comparison with available historical and screening images. The comparative analysis was used to determine the outcome of the chest examination. When the interpretations of these two radiologists were different, they need to analyze repeatedly and negotiated the judgment. In addition, at least two qualified respiratory medicine and chest tumor experts from our hospital formed a diagnosis team. Based on the recommendation of radiologists and other results of physical examination, the diagnosis team made a further plan of intervention treatment. LDCT scans that could reveal any non-calcified nodule with at least 4 mm diameter were classified as positive, suspected lung cancer. Other abnormalities such as obstructive atelectasis, soft tissue or patchy clouding opacity could be classified as a positive result as well.

### Medical-record abstraction

Data regarding diagnostic evaluation procedures and any associated complications for patients with positive screening tests and lung cancer were extracted from the medical records. At the same time, the pathology reports and records of operation and treatment of patients with lung cancer were also obtained. Metastatic lung cancer was excluded. The classification of histologic characteristics of the lung cancer were conducted according to the International Classification of Diseases for Oncology, 3rd Edition (ICD-O-3) [19], and the clinical stages were conducted according to the eighth edition of the Cancer Staging Manual of the American Joint Committee on Cancer (AJCC) [20].

### Vital status

We have trained professionals to follow-up. A special follow-up team is responsible for ascertaining probable vital status and determining whether the cause of death was lung cancer. We have carefully distinguished between lung cancer-related deaths and those caused by diagnostic evaluation or treatment of lung cancer.

### Statistical analysis

SPSS was adopted for statistical analysis. Comparisons between the group of patients with lung cancer and the group of patients without lung cancer were calculated by the chi-square test (categorical data) or the T-test (numerical data). *P* value < 0.05 was considered as statistically significant. Survival analysis was performed by Kaplan-Meier with ungrouped data.

## Results

### Characteristics of the participants

The 15,996 subjects completed baseline lung screening with LDCT from June 30, 2006 through June 30, 2017. The baseline demographic characteristics of the 15,996 participants are summarized in Table 1. The age of participants was distributed in all ages, but it was mainly over 40 years old. 11,247 (70.3%) individuals did not smoke.

**Table 1:** Selected Baseline Characteristics of the Study Participants

Parameter	Male, n (%)	Female, n (%)	Total, n (%)
Total	9801 (61.3)	6195 (38.7)	15,996 (100.0)
Age at inclusion (years)^a^	51.26 ± 14.76	48.80 ± 14.76	50.31 ± 14.81
< 30	545 (5.56)	586 (9.5)	1131 (7.0)
30~	1361 (13.9)	872 (14.1)	2233 (14.0)
40~	3119 (31.8)	2224 (35.9)	5343 (33.4)
50~	2363 (24.1)	1196 (19.3)	3559 (22.2)
60~	1033 (10.5)	605 (9.8)	1638 (10.2)
70~	832 (8.5)	475 (7.7)	1307 (8.2)
≥80	548 (5.6)	237 (3.8)	785 (5.0)
Smoking status			
smoking	4697 (47.9)	52 (0.8)	4749 (29.7)
non-smoking	5104 (52.1)	6143 (99.2)	11,247 (70.3)
Smoking volume (pack year)^a^	19.24 ± 14.84	13.95 ± 25.04	19.20 ± 14.93
< 10	894 (19.0)	13 (25.0)	907 (19.1)
10~	1080 (23.0)	8 (15.4)	1088 (23.0)
20~	841 (17.9)	1 (1.9)	842 (17.7)
30~	488 (10.4)	1 (1.9)	489 (10.3)
40~	225 (4.8)	0 (0)	225 (4.7)
50~	45 (1.0)	0 (0)	45 (0.9)
≥60	100 (2.1)	1 (1.9)	101 (2.1)
Unknown	1024 (21.8)	28 (53.8)	1052 (22.2)
Family history of lung cancer			
Yes	163 (87.2)	181 (84.6)	401 (2.5)
No	24 (12.8)	33 (15.4)	15,595 (97.5)
Chronic lung disease^b^			
Yes	5868 (59.9)	3450 (55.7)	9318 (58.3)
No	3933 (40.1)	2745 (44.3)	6678 (41.7)

^a^ Values are presented as mean ± SD (range). ^b^ Chronic lung diseases include the chronic obstructive pulmonary disease, diffuse pulmonary fibrosis, history of pulmonary tuberculosis and other respiratory diseases

### Positive results of screening

In the lung cancer screening with LDCT, positive results are shown in Table 2. Of the 15,996 participants with physical examination who completed the baseline screening, 6779 subjects had at least one positive finding, with a positive rate of 42.4% (Table 2).

**Table 2:** Results of Screening

Parameter	Health Examination
	Population
	n(%)
Total No. Screened	15,996
Positive Result	6779 (42.4)
Lung cancer confirmed	142 (2.1)
T0^a^	86 (1.9)
T1	31 (1.9)
T2	20 (3.9)
T3	4 (3.5)
T4	1 (4.2)
Lung cancer not confirmed	6637 (97.9%)
T0^a^	4441 (98.1)
T1	1574 (98.1)
T2	490 (96.1)
T3	109 (96.5)
T4	23 (95.8)
Negative Result	9217 (57.6)

^a^ The screenings were performed at 1-year intervals, with the first screening (T0) performed soon after the time of randomization

### Incidence of lung cancers

The detection rates of lung cancer are summarized in Table 2. A total of 142 cases of lung cancer were screened out from the health examination population initially recruited in this research, the detection rate of lung cancer was 2.1%, and the false positive rate was 97.9% (Table 2).

We conducted this analysis in high-risk population of lung cancer. The analysis showed that based on the guidelines of the lung cancer screening proposed by the USPSTF [5, 6], of the 142 cases of lung cancer, 72 patients were aged 55–80, and 70 patients did not meet the age-standard (Table 3). In addition, the proportion of patients who met the smoking and quit-smoking standards were 8.5 and 2.1% respectively (Table 3). Only 13 cases of lung cancer met all criteria of screening (Table 3). The rate of missed diagnosis was as high as 90.8% according to the guideline of the lung cancer screening proposed by the USPSTF (Table 3).

**Table 3:** The results of lung cancer screening according to the USPSTF screening guidelines

Parameter	Number of lung cancer (***n*** = 142)	
	Met the inclusion criteria, n(%)	Not met the inclusion criteria, n(%)
Age at inclusion (years)		
A. 55 ~ 80	72 (50.7)	70 (49.3)
Smoking volume (pack year)		
B. ≥ 30	12 (8.5)	130 (91.5)
Quit smoking time (years)		
C. < 15	3 (2.1)	139 (97.9)
AB or AC	13 (9.2)	129 (90.8)

Moreover, we did the similar research in high-risk population of lung cancer based on the Chinese screening guidelines. According to the guidelines of the lung cancer screening in China [7], of the 142 cases of lung cancer, the proportion of patients who met the standards, including age, smoking, tumor history and chronic lung diseases, was 53.5, 12.7, 18.3 and 14.7%, respectively (Table 4). Just 25.4% people with lung cancer met all criteria of this guideline. The rate of missed diagnosis was 74.7% (Table 4).

**Table 4:** The results of lung cancer screening according to the Chinese screening guidelines

Parameter	Number of lung cancer (***n*** = 142)	
	Met the inclusion criteria, n(%)	Not met the inclusion criteria, n(%)
Age at inclusion (years)		
A. 50 ~ 75	76 (53.5)	66 (46.5)
Smoking volume (pack year)^a^		
B. ≥ 20	18 (12.7)	124 (87.3)
History of cancer^b^		
C. Yes	26 (18.3)	116 (81.7)
Chronic lung disease^c^		
D. Yes	45 (14.7)	145 (85.3)
A(B or C or D)	36 (25.4)	106 (74.7)

^a^This includes current smoking and quitting smoking less than 15 years. ^b^This includes a history of cancer or a family history of lung cancer. ^c^This includes the chronic obstructive pulmonary disease, diffuse pulmonary fibrosis, history of pulmonary tuberculosis and other respiratory diseases

### Analysis of high-risk factors in lung cancer stratified by gender

Previous studies have shown that there were differences in the high-risk factors of lung cancer between different genders [10, 21, 22]. Therefore, we further investigated the high-risk factors of lung cancer by gender. As shown in Table 5, age and smoking status were high-risk factors of lung cancer in women, while in men, besides these factors, there were some other high-risk factors, such as COPD, diffuse pulmonary fibrosis, and previous history of malignant tumor (all *P* < 0.05). In addition, we found that the detection rate of lung cancer in women was 1.1%, significantly higher than that in men 0.7% (*P* = 0.021) (Table 5). In order to explore the correlation between different age stages and lung cancer, we further analyzed the risk factor of age (Table 5). The proportion of lung cancer in men under 50, 50–55, 55–75, 75–80 and over 80 years old were 23.2, 11, 42.5, 12.3 and 11.0% respectively, and those in women were 33.3, 14.5, 33.4, 8.7, 10.1% respectively (Table 5). At the same time, we also found that among patients with lung cancer in male, the proportion of smokers was 75%, and that in female lung cancer cases was only 5.8% (Table 5).

**Table 5:** Analysis of high-risk factors in lung cancer stratified by gender

Characteristic	Male			Female		
	Patients with	Patients without	***P*** value	Patients with	Patients without	*P* value
	lung cancer, n(%)	lung cancer, n(%)		lung cancer, n(%)	lung cancer, n(%)	
Total	73 (0.7)	9728 (99.3)		69 (1.1)	6126 (98.9)	0.021 ^b^
Age (years) ^a^	62.10 ± 13.51	51.18 ± 14.74	< 0.0001	59.17 ± 13.44	48.69 ± 14.73	< 0.0001
< 30	0 (0)	545 (5.6)		0 (0)	586 (9.6)	
30~	0 (0)	627 (6.4)		0 (0)	397 (6.5)	
35~	2 (2.7)	732 (7.5)		1 (1.4)	474 (7.7)	
40~	6 (8.2)	1497 (15.4)		8 (11.6)	1158 (18.9)	
45~	9 (12.3)	1607 (16.5)		14 (20.3)	1044 (17.0)	
50~	8 (11.0)	1288 (13.2)		10 (14.5)	710 (11.6)	
55~	4 (5.5)	1063 (10.9)		4 (5.8)	472 (7.7)	
60~	7 (9.6)	591 (6.1)		6 (8.7)	363 (5.9)	
65~	13 (17.8)	422 (4.3)		5 (7.2)	231 (3.7)	
70~	7 (9.6)	446 (4.6)		8 (11.6)	192 (3.1)	
75~	9 (12.3)	370 (3.8)		6 (8.7)	269 (4.4)	
80~	8 (11.0)	540 (5.6)		7 (10.1)	230 (3.8)	
Smoking history	55 (75.3)	5299 (54.5)	0.000	4 (5.8)	52 (0.8)	0.003
Chronic obstructive pulmonary disease	24 (32.9)	1821 (18.7)	0.002	9 (13.0)	606 (9.9)	0.384
Diffuse pulmonary fibrosis	17 (23.3)	1440 (14.8)	0.042	6 (8.7)	732 (11.9)	0.407
Previous pulmonary tuberculosis	4 (5.5)	357 (3.7)	0.346	1 (1.4)	168 (2.7)	1.000
Previous history of malignant tumor	11 (15.1)	268 (2.8)	< 0.0001	12 (17.4)	691 (11.3)	0.112
Family history of cancer	5 (6.8)	723 (7.4)	0.850	10 (14.5)	751 (12.3)	0.574
Number of first-degree relatives with cancer	5 (6.8)	653 (6.7)	0.371	10 (14.5)	655 (10.7)	0.478
Family history of lung cancer	2 (2.7)	185 (1.9)	0.651	1 (1.4)	213 (3.5)	0.733
Number of first-degree relatives with lung cancer	1 (1.4)	162 (1.7)	0.287	1 (1.4)	180 (2.9)	0.888

^a^Values are reported as mean ± SD (range). ^b^The detection rate of lung cancer in women is compared with that in men.

### Mortality

**Fig. 1:**
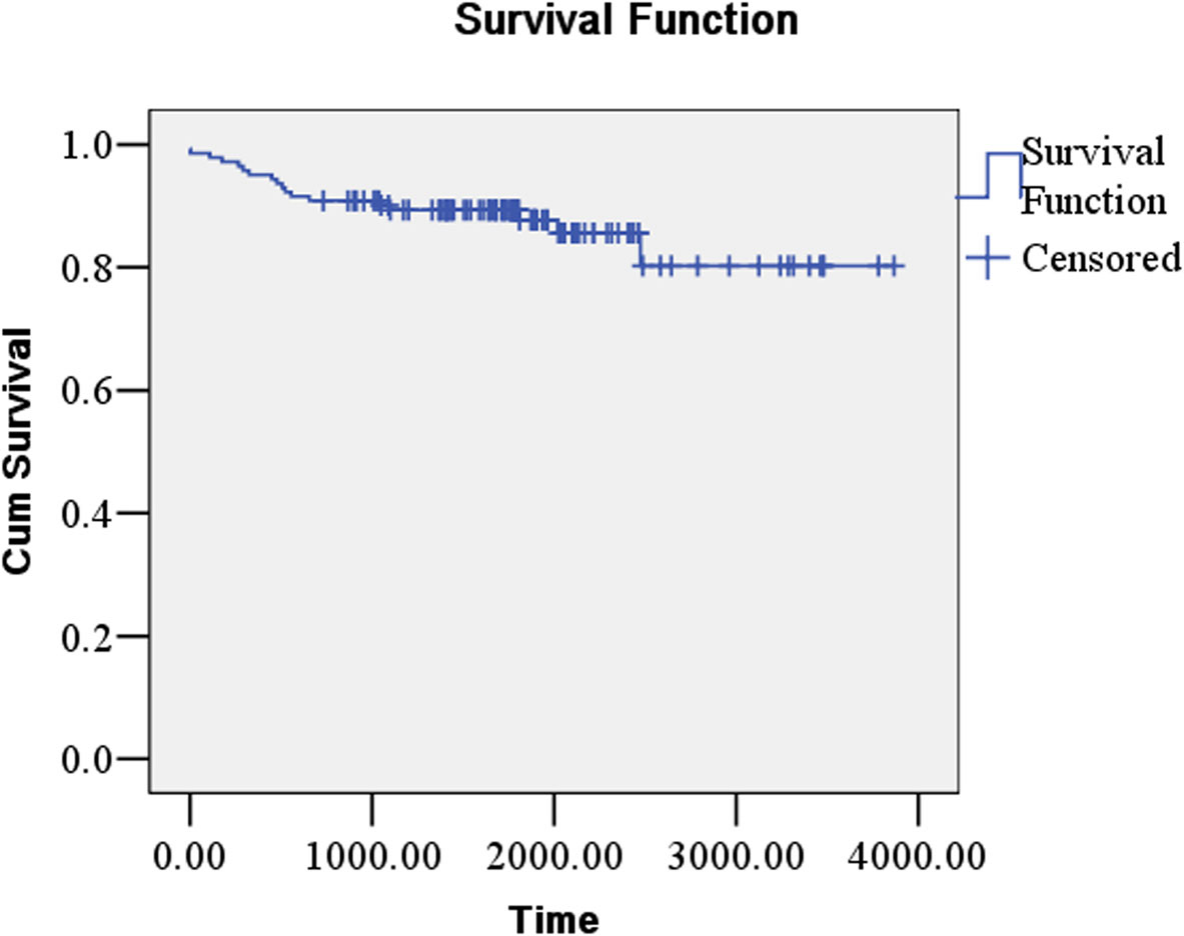
Survival curve of lung cancer patients

Participants were followed-up from January 1, 2007 to January 31, 2020. The shortest follow-up time was 2.5 years, and the longest was 13 years. Up to the end of follow-up, 963 people lost their visit, the rate of which was 6.0%. There were 18 deaths of all the patients with lung cancer. The mortality rate was 12.7%. Lung cancer accounted for 55.5% of all the deaths in the study (Table 6). In addition, we plotted survival curves for patients with lung cancer (Fig. 1).

**Table 6:** Causes of Death among Lung Cancer Patients

Cause of Death ^**a**^	Number/Total number (%)
Neoplasm of bronchus and lung	10/18 (55.5)
Respiratory illness	4/18 (22.2)
Multiple organ failure	3/18 (16.7)
Other	1/18 (5.6)

^a^Causes of death were categorized according to the codes in the International Classification of Diseases, 10th Revision (ICD-10)

## Discussion

The current lung cancer screening guidelines define high-risk groups in terms of age, smoking and so on [5–7, 23]. However, the high-risk factors of lung cancer are different between countries and regions [5–7, 23]. In order to know whether the current screening guidelines of lung cancer will cause missed diagnosis of lung cancer cases in the health examination population, we firstly conducted a real-world study using these screening guidelines in this Chinese population. Our results suggested that the current screening guidelines of lung cancer might miss some of the high-risk population in the health examination population. Several factors as following may contribute to this.

On the one hand, of 142 patients with lung cancer in our study, non-smokers accounted for 67.6%. In female patients with lung cancer, the proportion of non-smokers was even as high as 94.2%. Recently, a study in South Korea had reported that 84 (40.6%) out of 207 patients with lung cancer were never-smokers [24]. Moreover, a study in Japan also showed that 49.6% of patients with lung cancer occurring in never smokers might be missed if we only adopted the National Lung Screening Trial (NLST) criterion of smokers with ≥30 pack-years of smoking [25]. The different proportion of lung cancer in never smokers is probably due to different smoking prevalence of East Asian females with lung cancer (9.9% in South Korea, 17–25.6% in Japan, and 5.2% in China), which was significantly lower than that in Caucasian female patients (ranging from 53 to 91%) [26–33]. Therefore, this may be the main reason why the missed diagnosis rate of lung cancer in this study is higher than that in previous studies abroad [10, 26–34]. Although there were differences in the proportion of non-smoking patients with lung cancer in different countries, they all accounted for a high proportion. Therefore, according to the current lung cancer screening guidelines, this part of lung cancer cases will be missed diagnosis, especially female patients. Researches have shown that the incidence of lung cancer is related to many factors including genetic factors, smoking, environmental exposure, air pollution, and so on [9, 35–42]. Generally, the vast majority (80%) of lung cancer cases are attributable to tobacco smoking [43, 44]. However, the global statistics estimate that 25% of all lung cancer cases worldwide are not due to smoking [10]. With the successful implementation of prevention and cessation programs of smoking, the proportion of lung cancer in non-smokers is expected to increase [8]. In addition, the epidemiological differences in lung cancer incidence and risk factors between Chinese and US populations suggest that inclusion of ambient air pollution exposure and gender into lung cancer risk prognostic models might better capture high-risk individuals, especially for non-smoking women [45]. Recently, a predicting lung cancer occurrence in never-smoking Asian females has been proposed [46].

On the other hand, of all lung cancers detected in our study, 28.1% patients were younger than 50 years, and 10.6% patients were older than 80 years. After further analysis by age and gender, we found that according to the age standard of lung cancer screening guidelines in China, the proportion of patients with lung cancer in men who met the criterion was 53.5%, and that in women was 47.9%. In other words, according to the Chinese standards, the proportion of male and female patients with lung cancer missed diagnosis were 46.5 and 52.1% respectively. In addition, according to age standard of lung cancer screening guidelines recommended by USPSTF, the proportion of lung cancer cases in male who met the criterion was 54.8%, and that in female was 42.1%. That is to say, based on the American Standards, the proportion of lung cancer cases missed diagnosis in men and women were 45.2 and 57.9% respectively. It can be seen that no matter which lung cancer screening guidelines you choose, there were many patients with lung cancer missed according to the age standard, especially female patients. Previous studies showed that the incidence of lung cancer in young adults were around 1.2 to 6.2% (less than 40 years), 5.3% (under 45 years), and 13.4% (less than 50 years) [47–51]. Therefore, with an increasing incidence of lung cancer in young people, it have gradually become a disease group that can not be ignored [52, 53].

In addition, our study found that compared with patients with lung cancer in male, female patients with lung cancer have the following characteristics. Firstly, the detection rate of lung cancer in women is higher than that in men. Secondly, the prevalence of non-smoking lung cancer in women was much higher than that in men. Previous studies have also found that the proportion of women with non-smoking lung cancer is higher than that of men [10]. Global statistics estimate that 53% of lung cancers in female and 15% in male are not attributable to smoking [10]. Thirdly, the proportion of young people among female patients with lung cancer is higher than that among male patients with lung cancer. The feature in our study is consistent with earlier result reported in previous studies [21, 22].

Meanwhile, the result showed that there was a high rate of false positive in lung cancer screening with LDCT in our study. This was consistent with the results of the National Lung Screening Trial (NLST) [4]. Most of the false positive results might be due to the presence of non-calcified granulomas or benign intrapulmonary lymph nodes.

To our knowledge, this is the first real-world study to explore whether the current lung cancer screening guidelines are applicable to the health examination population in China. The missed diagnosis rate is very high whether using American or Chinese screening guidelines of lung cancer. Moreover, our study was a real-world cohort study with large sample size. The results could reflect the real clinical situation comprehensively and accurately. In this way, we could fully understand the gap between the guidelines and practice, and provide reference for the formulation and specification of the guidelines.

Our research also has some limitations. First, the study population mainly came from Chengdu and surrounding areas. Relevant research that includes other regions is needed. Second, our research population is mainly the staff of enterprises and institutions. Personnel structure is relatively single. We need to further enrich our personnel structure to reflect the situation more realistically. Finally, the follow-up time is too short to calculate the 5-year and 10-year survival rates together. Thus, we should extend the follow-up time to know the long-term survival rate of lung cancer.

At present, the wide-spread clinical implementation of LDCT is hampered by clinical and socio-economic limitations, and access and adherence to LDCT screening programmes in high-risk populations remains low [54]. New, more accessible screening methods might improve uptake and adherence. Recently, emerging data on the role of liquid biopsy in early-stage NSCLC suggested that ctDNA analyses might allow lung cancer detection and could be potentially integrated in currently screening programs [55].

## Conclusions

This real-world study is the first research applied the current lung cancer screening guidelines to the health examination population in China. The results suggested that the rate of missed diagnosis was very high. In addition, our study also found that female patients with lung cancer have some characteristics when compared with patients with lung cancer in male. As shown in our data, further study to determine screening guidelines for targeted populations, is warranted.

## Data Availability

The datasets used and/or analyzed during the current study are available from the corresponding author on reasonable request.

## Abbreviations

USPSTF: U.S. preventive services task force
LDCT: Low-dose computed tomography
CT: Computed tomography
COPD: Chronic obstructive pulmonary disease
ICD-O-3: International classification of diseases for oncology, 3rd edition
AJCC: American joint committee on cancer
NLST: National lung screening trial
